# Quality Control of Structural MRI Images Applied Using FreeSurfer—A Hands-On Workflow to Rate Motion Artifacts

**DOI:** 10.3389/fnins.2016.00558

**Published:** 2016-12-06

**Authors:** Lea L. Backhausen, Megan M. Herting, Judith Buse, Veit Roessner, Michael N. Smolka, Nora C. Vetter

**Affiliations:** ^1^Department of Child and Adolescent Psychiatry, Faculty of Medicine of the Technische Universität DresdenDresden, Germany; ^2^Department of Preventive Medicine, University of Southern California, Los AngelesLos Angeles, CA, USA; ^3^Department of Psychiatry and Neuroimaging Center, Technische Universität DresdenDresden, Germany

**Keywords:** structural MRI, quality control, head motion, attention-deficit/hyperactivity disorder (ADHD), rating system, volumetry

## Abstract

In structural magnetic resonance imaging motion artifacts are common, especially when not scanning healthy young adults. It has been shown that motion affects the analysis with automated image-processing techniques (e.g., FreeSurfer). This can bias results. Several developmental and adult studies have found reduced volume and thickness of gray matter due to motion artifacts. Thus, quality control is necessary in order to ensure an acceptable level of quality and to define exclusion criteria of images (i.e., determine participants with most severe artifacts). However, information about the quality control workflow and image exclusion procedure is largely lacking in the current literature and the existing rating systems differ. Here, we propose a stringent workflow of quality control steps during and after acquisition of T1-weighted images, which enables researchers dealing with populations that are typically affected by motion artifacts to enhance data quality and maximize sample sizes. As an underlying aim we established a thorough quality control rating system for T1-weighted images and applied it to the analysis of developmental clinical data using the automated processing pipeline FreeSurfer. This hands-on workflow and quality control rating system will aid researchers in minimizing motion artifacts in the final data set, and therefore enhance the quality of structural magnetic resonance imaging studies.

## Introduction

For structural magnetic resonance imaging (sMRI), quality control (QC) of the T1-weighted images is essential due to artifacts possibly biasing results (Reuter et al., 2015). This includes technical artifacts like head coverage, radiofrequency noise, signal inhomogeneity, and susceptibility, as well as motion artifacts like blurring and ringing (Wood and Henkelman, 1985; Reuter et al., 2015). Motion artifacts are produced by the participant swallowing, blinking, chewing, turning, fidgeting, or repositioning a limb (Bellon et al., 1986). MRI technologists or physicists should care about technical artifacts, while motion artifacts require the attention of researchers. Therefore, researchers should be informed about different types of artifacts and their impact on the data. Motion artifacts are especially a problem in developmental studies (Brown et al., 2010; Van Dijk et al., 2012) with younger age groups related to increased motion artifacts (Blumenthal et al., 2002). Moreover, images of children and adolescents with psychiatric disorders, such as attention-deficit/hyperactivity disorder (ADHD), tic disorders (Buse et al., 2016), autism spectrum disorder, schizophrenia (Pardoe et al., 2016), and conduct disorder (CD, Huebner et al., 2008) might be particularly prone to motion artifacts. For example, ADHD impulsivity and hyperactivity symptoms have been shown to relate to more severe motion artifacts (Rauch, 2005).

The first important approaches to reduce motion artifacts are prospective techniques. Preparing participants with a mock scanner alone (Epstein et al., 2007) or in combination with motion-reduction training (Slifer et al., 2002) can help to acclimatize the child to the scanner environment and decrease children's anxiety (Törnqvist et al., 2006). Other approaches include providing clear instructions to the child to remain still (Kuperman et al., 2011; Van Dijk et al., 2012), using equipment for head fixation (Overmeyer et al., 2001; Shaw et al., 2007; Reuter et al., 2015), presenting a movie during the scan (Overmeyer et al., 2001), or scanning in the evening to promote natural sleep (Blumenthal et al., 2002; Shaw et al., 2007). For a more detailed description of prospective motion-reduction techniques see Woods-Frohlich et al. (2010).

Despite these efforts, however, developmental populations often struggle with keeping still during scanning. Thus, in addition to prospective motion-reduction techniques, retrospective QC remains necessary to rule out distortion due to motion artifacts (Blumenthal et al., 2002; Gedamu, 2011). For example, large motion artifacts have been shown to affect segmentation and parcelation techniques such as the automated image-processing pipeline FreeSurfer (Reuter et al., 2015; Tisdall et al., 2016). Volume and thickness estimates of cortical gray matter (GM) are biased by motion. A small increase in motion accounted for around 1.4–2.0% GM volume loss in an adult population, which is comparable to yearly atrophy rates in neurodegenerative diseases (Reuter et al., 2015). In a child population, Blumenthal et al. (2002) also found that there was a dose-dependent effect of motion artifacts and estimated GM volume loss, with mild motion associated with 4%, moderate motion associated with 7%, and severe motion associated with 27% reduction of total GM. For these reasons, previous studies including developmental clinical populations (such as ADHD) have had to exclude 4–23% of participants due to severe motion artifacts (Castellanos et al., 2002; Shaw et al., 2007; Huebner et al., 2008; Lopez-Larson et al., 2012).

Although most developmental and clinical sMRI studies exclude participants due to excess motion, to our knowledge, there is no established threshold or criterion for this “critical level” of motion. This is in contrast to functional MRI (fMRI) where techniques such as “spikes > 3 mm” after automated realignment preprocessing or thresholds based on “scrubbing” (for review see Power et al., 2015) are used to exclude participants due to motion. Without such automated algorithms, qualitative QC is required for each participant. Surprisingly, only some developmental sMRI studies report details of their retrospective QC approach. Based on evaluated literature search up to June 2015 of 57 studies found on sMRI in developmental ADHD and CD, only 10 reported some kind of retrospective QC. Of those, the approaches differ and are often reported without any details. For example, some authors note that T1-weighted images have been “checked for scanner artifacts and gross neuroanatomical abnormalities” (Fairchild et al., 2011, 2013), others merely state images have been “quality controlled for motion” (Dirlikov et al., 2015), or that they underwent “visual inspection” (Castellanos et al., 2002; Cao et al., 2010) or “internal quality control” (Fjell et al., 2015).

A few QC rating systems for motion artifacts in T1-weighted images do exist. These rating systems include categories ranging from “good” data, which is proposed to be included in further processing, to “moderate” data, and finally “bad” data, which should be excluded from further processing (Blumenthal et al., 2002; Wilke et al., 2002; Shaw et al., 2007; Pardoe et al., 2016; Reuter et al., 2015; Tisdall et al., 2016). However, the definition and range of additional categories in the “moderate” category, between the good and bad data categories, varies in previous work (Blumenthal et al., 2002; Shaw et al., 2007; Reuter et al., 2015; Tisdall et al., 2016). For instance, some authors used a 4-point scale (from none to severe, Blumenthal et al., 2002; Reuter et al., 2015) while others used a 5-point scale (from no detectable motion to lowest quality/severe motion, Pardoe et al., 2016). Moreover, most authors to date that have reported using QC ratings have not specified which artifact type(s) they focused on (e.g., ringing, blurring, gray, and white matter differentiation etc.) to evaluate motion in their images (Blumenthal et al., 2002; Wilke et al., 2002; Shaw et al., 2007; Pardoe et al., 2016). Reuter et al. (2015) are an exception, as they indicated that their rating system was based on artifacts like head coverage, wrapping, radiofrequency noise, signal inhomogeneity, susceptibility, and ringing. Specifically, they rated these artifact types from 1 to 4 and then merged these ratings into an overall quality category, i.e., either “pass,” “warn,” or “fail.” Using this approach, they found that cortical GM was significantly reduced for adult participants rated “fail” but also for those rated “warn,” suggesting participants in the “fail” and probably also in the “warn” category should be excluded from further analyses. However, the large number and range of images that fall into the “warn” category suggests that the QC rating system of Reuter et al. (2015) may need to be adapted and refined in order to save as much data as possible and obtain reliable statistical results at the same time.

A standard and robust retrospective QC rating system is warranted to improve replication and comparability between studies and to ensure that only participants with an acceptable level of image quality contribute to the results. Thus, the aim of the current study was two-fold. First, we aimed to propose a stringent workflow of QC steps of sMRI T1-weighted images in detail, which especially enables developmental researchers or those doing group comparisons, especially with patient groups, to efficiently process valuable data. At the same time, we aimed to establish a thorough qualitative QC rating system for T1-weighted images to train research team members on motion and other artifacts before the start of a study and to rate images retrospectively. As our second aim we implemented and tested this rating system in a developmental clinical sample. We sought to replicate previous findings that motion artifacts influence GM volume estimates. Overall, we tested if our rating system captures biases due to motion artifacts. For future studies, the application of the proposed hands-on workflow and qualitative QC rating system may further help to minimize critical motion artifacts in the final data set used for statistical analyses, and therefore boost the quality of future sMRI studies.

## Materials and methods

As our first aim, we developed a workflow including a T1 rating system and applied it to our developmental clinical data. As a second aim we tested if motion artifacts in our data influence the estimation of GM volume (see Reuter et al., 2015). Therefore, we applied our rating system on data of clinical and typically developing (TD) adolescents.

### First aim: workflow including T1 rating

#### T1 rating system

**Figure 1:**
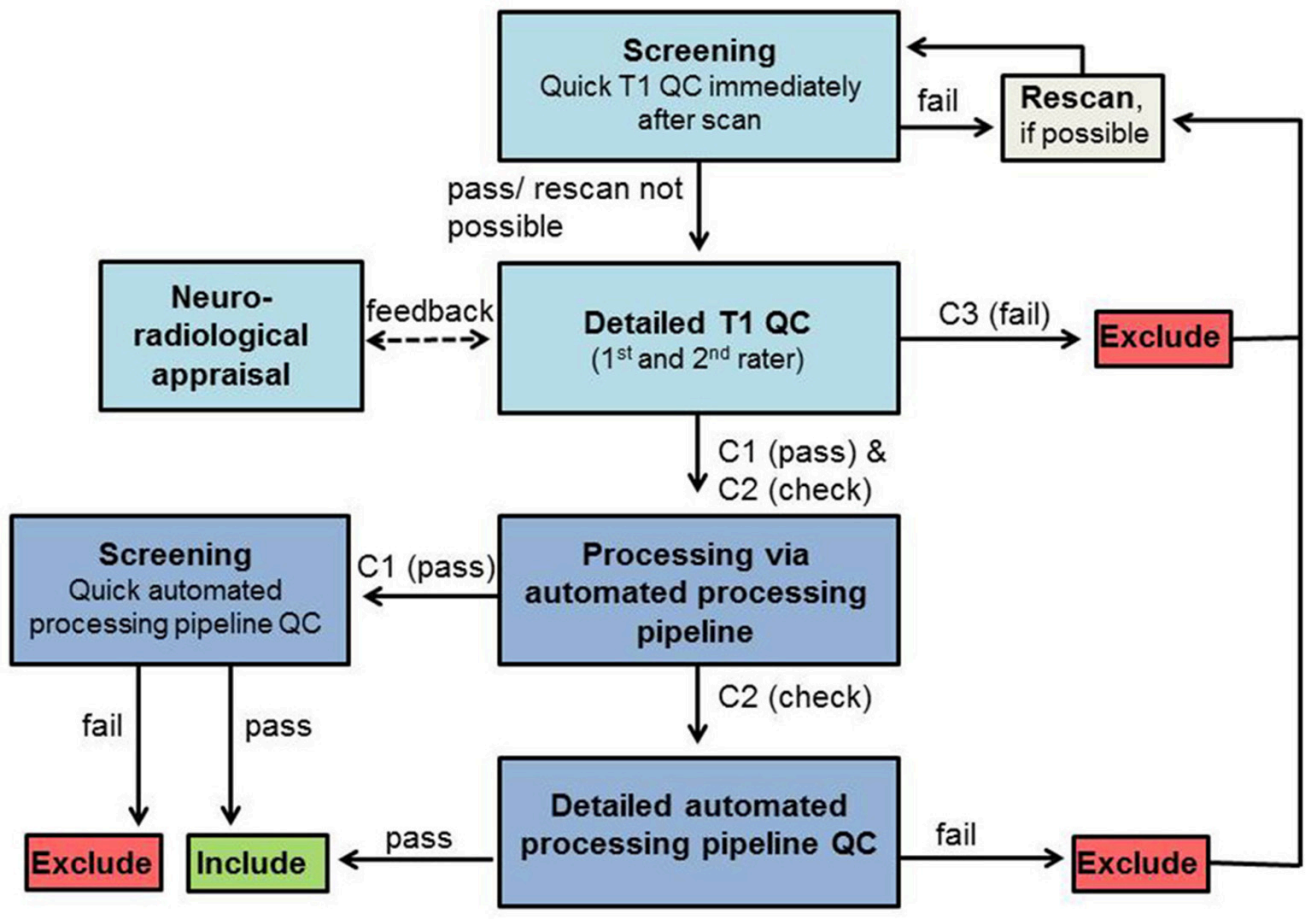
**QC workflow**. Boxes in light blue represent QC steps using T1-weighted images and boxes in dark blue represent QC steps using processed images via automated processing pipeline. Exclude/Include: exclusion/inclusion of data set in further preprocessing and analysis.

**Figure 2:**
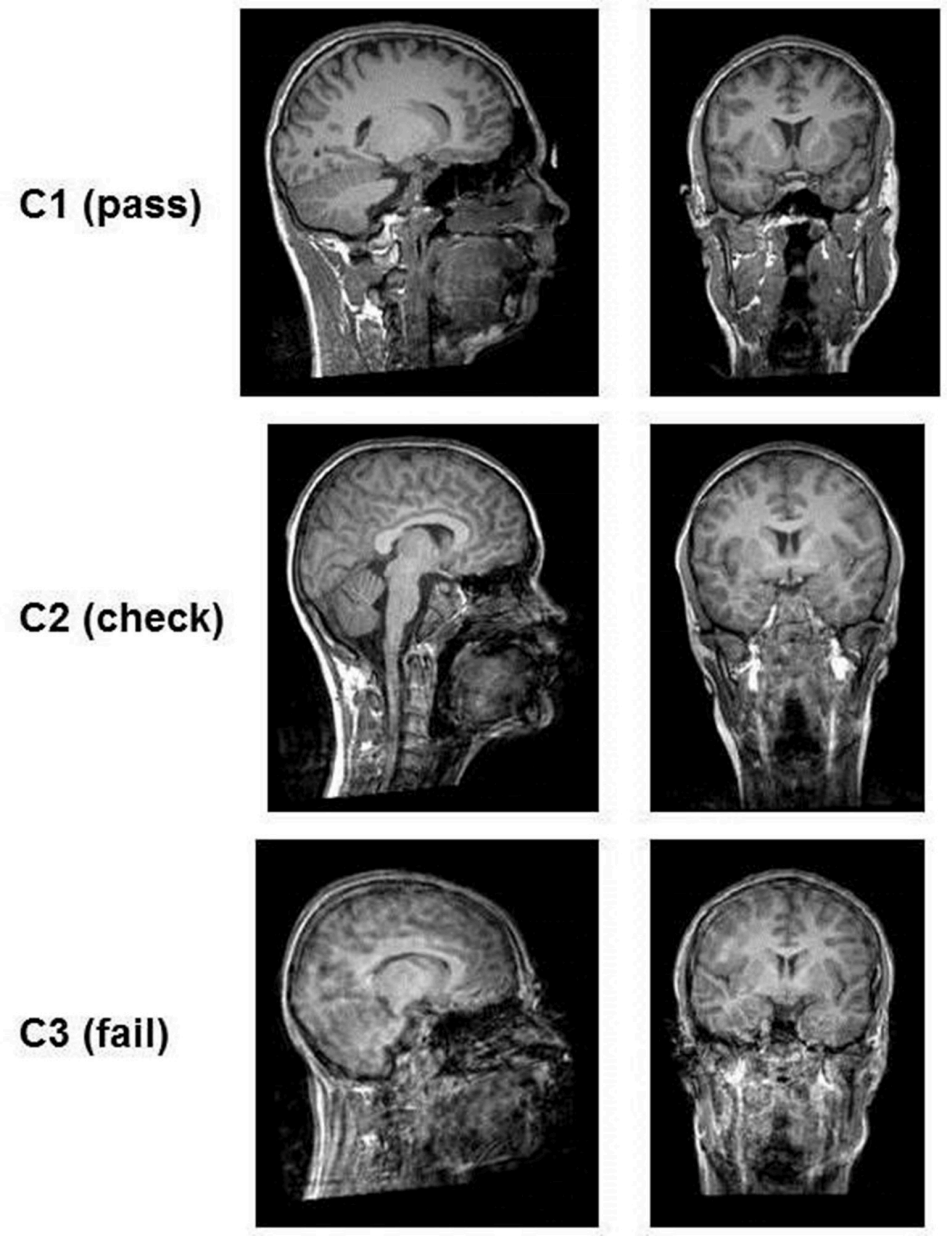
**Examples of the quality of T1-images according to rating categories**. The images in category C1 (pass) are clear, no motion artifacts or ringing can be seen and subcortical structures as well as gray matter/white matter can be well differentiated. In category C2 (check) ringing can be seen but the contrast of the structures themselves is good and they can be differentiated. In category C3 (fail) ringing as well as motion artifacts (distortion) are present. The contrast is very poor and structures blend into each other.

This qualitative rating system was developed to visually rank the quality of T1-weighted images taking into account the three artifacts most present in our developmental clinical data, including motion, ringing, and susceptibility (for a definition of these artifacts see Supplementary Material). Ringing was seen in ~40% of T1-weighted images. These artifacts have been previously focused on in QC analyses (Blumenthal et al., 2002; Shaw et al., 2007; Reuter et al., 2015; Pardoe et al., 2016). Four different rating categories were chosen because some artifacts can affect different components of an image. For example, ringing artifacts tend to only affect GM and white matter (WM) borders but not subcortical structures (Table 1). The benefit of this approach is the possibility to focus the rating on certain areas according to the study question. The first two steps concern artifacts like ghosting, blurring or susceptibility artifacts (step 1: “Image sharpness”) and ringing (step 2: “Ringing”), the other two apply to how well-crucial information can be drawn from the image [step 3: “Contrast to noise ratio (CNR) (subcortical structures),” step 4: “CNR (GM and WM)”]. First of all, ratings from R1 to R3 are assigned to each step. The mean score of these four rating steps is then calculated and represents the final category of C1 (pass), C2 (check) or C3 (fail). Depending on the study's focus, the different steps can be weighted when calculating the average; such as step 3 of subcortical structures could have a higher impact on the final score/category. These final category assignments can then be used to decide whether to include or exclude images from further analyses (see Figure 1). T1-weighted image examples for each of the categories are presented in Figure 2.

**Table 1:** **Rating system structure**.

Step 1: Image sharpness	R1 (good): Clear/rather clear image; ghosts, blurred regions, or other artifacts if at all minor; no susceptibility artifacts
	R2 (moderate): Rather coarse/blurred image; moderate motion artifacts; if susceptibility artifacts are present they do not influence relevant areas
	R3 (bad): Obviously coarse/blurred image; major motion and susceptibility artifacts (e.g., due to dental braces)
Step 2: Ringing	R1 (good): No/slight ringing artifacts seen; at most in one region
	R2 (moderate): Ringing artifacts in more than one region
	R3 (bad): Circular ringing artifacts throughout the whole image
Step 3: CNR (subcortical structures)	R1 (good): Sharp edges; structures can be well-identified
	R2 (moderate): Structures still can be identified but less clear
	R3 (bad): Structures can hardly be identified
Step 4: CNR (GM and WM)	R1 (good): Sharp edges; GM and WM are well-differentiated
	R2 (moderate): GM and WM not well-differentiated
	R3 (bad): Borders of GM and WM blend; not differentiated at all

*CNR, contrast to noise ratio; GM, gray matter; WM, white matter*.

The complete workflow used in our developmental clinical sMRI study and the implementation of our QC rating system is presented in Figure 1.

#### Workflow

##### Screening

Immediately after data acquisition T1-weighted images should be screened by the trained experimenter on the MRI console to identify obvious artifacts and potentially rescan the participant, again reminding them of the importance to stay still, to prevent rating categories C3 (and C2 depending on the study protocol and time restrictions). For practical reasons, we used the usual scanner software (Siemens Magnetom) for this first visual check. The image was checked full screen (22.0″) when scrolling through the sagittal plane only to assess its overall quality (i.e., without focusing on any specific anatomical landmarks). This allows for a sufficient assessment of the image and the decision as to whether rescanning is necessary.

##### T1 QC

After data collection, the quality of each T1-weighted image is visually rated from C1 (pass) to C3 (fail), blind to group/patient information in order to prevent biased ratings. This QC of T1-weighted image can be done in any NIfTI format viewer like MRIcron. Contrast settings should be set similar for all images. While rating each step, it is important to scroll through all slices to get a good impression about the image quality. Additionally, a neuroradiologist should be consulted to check for gross/clinically relevant anatomical alterations. These alterations might lead to data exclusion depending on the study aim and severity of alterations. All images rated C3 (fail) have to be excluded from further analyses. Participants with images rated C3 (fail) can be invited again later for a rescan to receive good quality data.

##### Automated processing pipeline

T1-weighted images rated C1 (pass) and C2 (check) are then processed by using the automated processing pipeline (e.g., FreeSurfer).

##### Automated output QC

Data from images rated C1 (pass) should be quality controlled shortly after the automated processing pipeline procession (screening) with focusing on deformation of the 3D brain anatomy and large truncated brain areas only (Ducharme et al., 2016). For C2 (check) a detailed automated processing pipeline QC is mandatory to double-check data falling into this category. For this C2 (check) data, the results (in case of FreeSurfer for segmentation: “aseg” and for parcellation “aparc”) are visually evaluated (in case of FreeSurfer using “Freeview”). They should be compared to the T1-weighted images with a specific focus on the previously detected artifacts to validate the automated processing pipeline results. For the C2 (check) images the automated processing pipeline QC focuses on the following issues that all have to pass to further be included in the analyses: (1) the deformation of the 3D brain anatomy and large truncated brain areas (Ducharme et al., 2016); (2) the removal of non-brain tissue, i.e., “skullstrip” (tested by overlapping the original T1-weighted image after intensity normalization named T1.mgz and the T1.mgz after skull stripping, named brainmask.mgz); (3) the plausibility of subcortical/cortical structure borders (tested by overlapping aseg.mgz, i.e., the color map of segmented subcortical structures) or aparc.mgz (i.e., the color map of segmented cortex) and brainmask.mgz); and (4), absence of any GM misclassification as (very dark) WM, so-called WM hypointensities (Tang et al., 2013). Once again, rating should be done blind to group/patient information to reduce bias.

### Second aim: implementation and test of our T1 rating system

To test whether our rating system has an impact on GM volume (see Reuter et al., 2015) in a developmental population, we applied the established rating system to analyze data of participants from three clinical types: (1) ADHD, (2) ADHD comorbid with CD (ADHD + CD), and (3) TD adolescents. All T1-weighted images were rated by two independent trained raters to establish inter-rater reliability as well as the ability to discuss critical cases. All ratings were done within 1 week.

#### T1 data acquisition

The acquisition of T1-weighted images was part of a study about emotion processing in ADHD, which included one fMRI paradigm before, and one after, the T1-weighted scan. Thirty-eight male adolescent ADHD patients (*M* = 14.14 ± 1.8 years), 23 male comorbid ADHD + CD patients (*M* = 12.82 ± 1.24 years) and 27 TD male adolescents (*M* = 14.47 ± 1.69 years) were recruited. Patients were diagnosed with hyperkinetic disorder, attention deficit disorder without hyperactivity, or hyperkinetic conduct disorder according to the ICD-10 (World Health Organization, 1992) by licensed psychologists. If on treatment, patients withdrew their stimulant medication 3 days before the fMRI assessment. The study was carried out according to the latest version of the Declaration of Helsinki and was approved by the local ethics committee. Both participants and parents or legal guardians, respectively, gave their written informed consent.

3D T1-weighted magnetization-prepared rapid gradient echo (MPRAGE) image data sets were acquired (TR = 1900 ms, TE = 2.26 ms, FOV = 256 × 256 mm, 176 slices, 1 × 1 × 1 mm voxel size, flip angle = 9°) using a 3T whole-body MR (Magnetom TRIO, Siemens, Dresden, Germany) equipped with a 12-channel head coil. The time required for each scan acquisition was 6 min. The prospective motion-reduction techniques used to minimize movement during MRI included having participants complete a mock scanner session, if desired, and given time for questions. We also aimed to motivate participants by telling them it only takes 6 min with a following pause and reminding them of rewards. Prior to scanning, foam padding was also placed around the head and participants were reminded of the importance to lay still. During the scan, participants could either close their eyes or look at a message on the screen again reminding them not to move.

#### Data procession (FreeSurfer)

Automated segmentation of subcortical structures (aseg) was performed with the FreeSurfer image analysis pipeline (Version 5.1), which is documented and freely available for download online (http://surfer.nmr.mgh.harvard.edu/). The processing includes removal of non-brain tissue, automated Talairach transformation and segmentation of the subcortical WM and deep GM volumetric structures. The last step is completed by automatically assigning one of 37 neuroanatomical labels to each voxel in the MRI volume based on probabilistic information from a manually labeled training set (Fischl et al., 2002). This method has been shown to be comparable to much slower, labor-intensive manual labeling methods (Fischl et al., 2002).

#### Statistical analyses and reliability

All statistical analyses were performed with SPSS (IBM SPSS Statistics for Windows, Version 21.0 Armonk, NY, USA). One-way between subjects analyses of variance (ANOVAs) were conducted to compare volume differences between QC rating categories in volume estimates derived from FreeSurfer.

## Results

Concerning our first aim, we computed the intra-class correlation coefficient of categories C1–C3 for two independent raters (two-way mixed model, type absolute agreeing). The average measures coefficient yielded excellent results (α = 0.931). The rating distribution of one trained rater for each group is shown in Table 2.

**Table 2:** **Rating distribution of all available T1-weighted images (***n*** = 88 in total)**.

**Rating category**	**ADHD (*n* = 38)**	**ADHD + CD (*n* = 23)**	**TD (*n* = 27)**	**Total (*n* = 88)**
C1 (pass)	31 (81.6%)	16 (69.6%)	21 (77.8%)	68 (77.3%)
C2 (check)	3 (7.9%)	4 (17.4%)	4 (14.8%)	11 (12.5%)
C3 (fail)	4 (10.5%)	3 (13.0%)	2 (7.4%)	9 (10.2%)

*ADHD, patients with attention-deficit hyperactivity disorder; ADHD + CD, ADHD patients with comorbid conduct disorder; TD, typically developing*.

**Figure 3:**
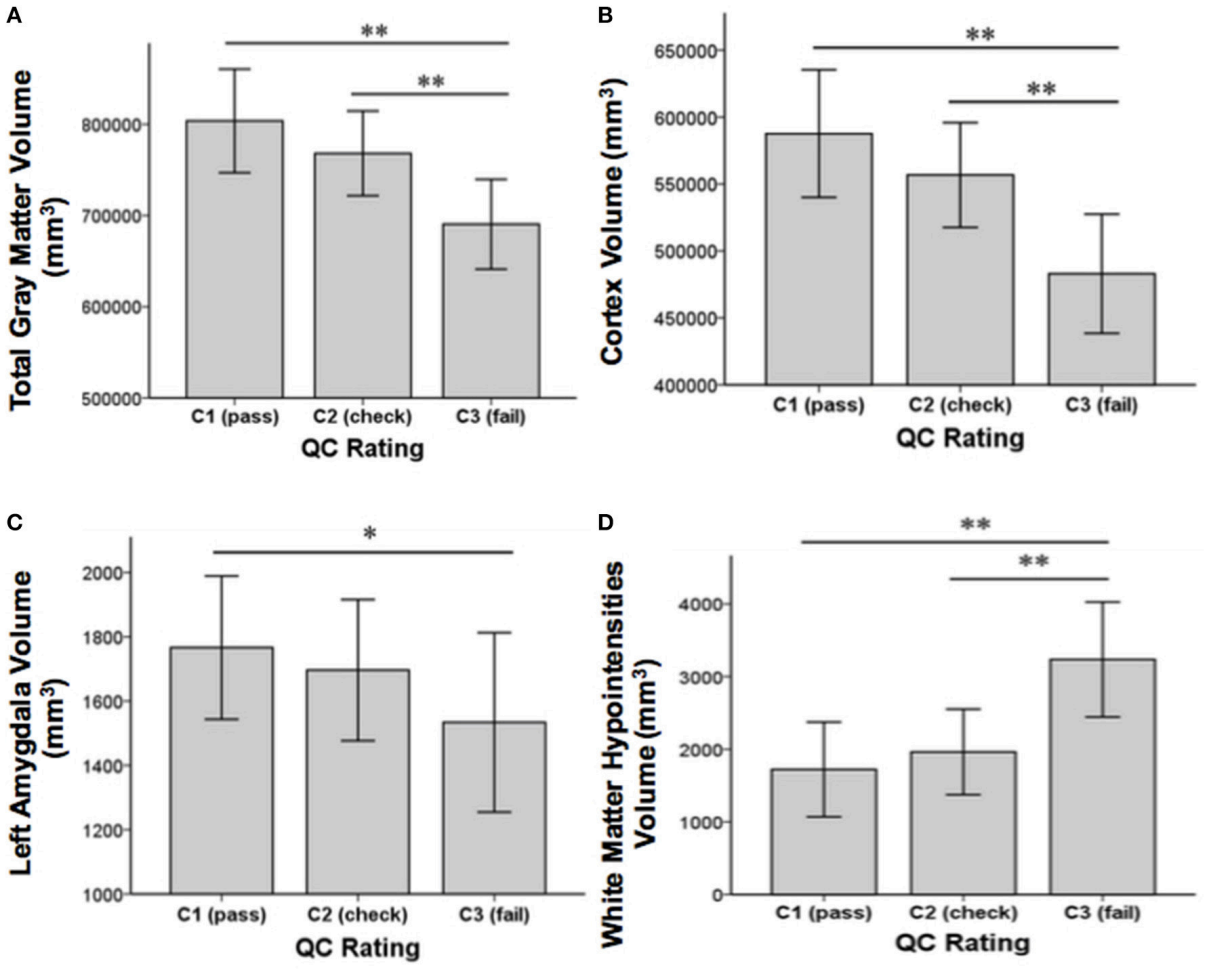
**Structural volume differences across QC rating categories for total gray matter (A)**, cortex **(B)**, left amygdala **(C)**, and white matter hypointensities **(D)**. Significance values refer to *post-hoc t*-tests. ^**^p < 0.01, ^*^p < 0.05. Error bars denote *SEM*.

Concerning our second aim, GM volume differences between the QC rating categories were found in the cortex [*F*_(2, 85)_ = 21.01, *p* < 0.001], the left caudate [*F*_(2, 85)_ = 7.26, *p* = 0.001], the left amygdala [*F*_(2, 85)_ = 4.29, *p* = 0.017], and total GM [*F*_(2, 85)_ = 17.65, *p* ≤ 0.001]. Additionally, differences between QC rating categories were found in WM hypointensities volume [*F*_(2, 85)_ = 20.98, *p* < 0.001]. Results still hold when controlling for group using analyses of covariance (ANCOVAs). *Post-hoc t*-tests revealed significant differences between QC rating categories C1 and C3 in GM volume of the cortex [*t*_(75)_ = 6.24, *p* < 0.001], total GM [*t*_(75)_ = 5.7, *p* < 0.001], the left caudate [*t*_(75)_ = 3.72, *p* < 0.001], and the left amygdala [*t*_(75)_ = 2.86, *p* = 0.006] as well as between C2 and C3 in the cortex [*t*_(18)_ = 3.94, *p* = 0.001], total GM [*t*_(18)_ = 3.63, *p* = 0.002], and the left caudate [*t*_(18)_ = 3.72, *p* = 0.002]. Volumes in categories C1 and C2 were bigger than those of category C3 in each case (see Figure 3). For WM hypointensities *post-hoc t*-tests revealed significant volume differences between QC categories C1 and C3 [*t*_(75)_ = −6.38, *p* < 0.001] as well as C2 and C3 [*t*_(18)_ = −4.13, *p* = 0.001] showing bigger volumes in category C3 than categories C1 and C2 (see Figure 3). The percentage GM volume loss and accordingly WM hypointensities volume gain in categories C2 and C3 compared to C1 were calculated for all structures which yielded significant results in the ANOVA. In category C2, we found volume losses of 5.3% for cortex, 4.4% for total GM, 4% for the left amygdala, 0.8% for the left caudate, and volume gain of 13.9% for WM hypointensities. In category C3, we found volume losses of 17.8% for cortex, 14.1% for total GM, 13.16% for the left amygdala, and 19% for the left caudate and volume gain of 87.9% for WM hypointensities.

Since age has been reported to correlate with motion (Blumenthal et al., 2002), we also explored a possible correlation of age with QC category. However, no significant correlation between age and QC category was found.

A chi-square test of independence was performed to examine the relation between group (ADHD, ADHD + CD and TD) and rating category (C1–C3). The relation between these variables was not significant, *X*^2^ (4, *N* = 88) = 1.861, *p* = 0.761, suggesting that the amount of motion artifact was not related to group membership in the current sample.

## Discussion

### Overview

This study aimed to develop a hands-on workflow for identifying and rating sMRI motion artifacts. First, we presented a stringent workflow of QC steps of T1-weighted images and described our detailed qualitative rating system. Next, we applied this rating system in a developmental clinical sample and tested for the influence of motion artifacts on FreeSurfer GM volume estimates. As expected, we found GM volume reduction in total GM, cortex, and some subcortical structures due to motion artifacts. These results underline the importance to assess movement in sMRI analyses.

This study is one of the first to establish a hands-on QC workflow and a qualitative rating system to minimize motion bias in sMRI results.

The proposed QC rating system yielded excellent inter-rater agreement. Furthermore, all images could easily and readily be assigned to one of the three rating categories. The three categories C1 (pass), C2 (check), and C3 (fail) provide useful information that is needed to decide whether to in- or exclude participant data. Images falling into category C3 (fail) were indeed of bad quality and thus should be excluded from further analyses. For four of the 13 C3 (fail) rated, FreeSurfer was unable to complete all data processing steps, which strongly indicates poor original T1-weighted data. These findings suggest that the currently presented QC rating system is able to correctly identify bad quality data. More importantly, the need to identify and exclude poor images is further highlighted by the results seen from comparing volume estimates and misclassifications between the different rating categories. Volume estimates in cortex, total GM, the left amygdala and the left caudate were significantly larger in C1 (pass) and C2 (check) categories as compared with C3 (fail). Similarly, percentage of GM volume reduction was more striking in category C3 (fail) than in category C2 (check). Misclassification (WM hypointensities) was also found to be more prominent in category C3 (fail) than in category C2 (check). The resulting percentage volume differences between motion categories for total GM (4.4% for category C2 and 14.1% for category C3) are similar to previous findings (Blumenthal et al., 2002; Reuter et al., 2015). Likewise, the volume reductions between motion categories were primarily driven by cortical volume. We also found significant motion artifact related differences in the left amygdala and the left caudate. These findings are similar to Pardoe et al. (2016), who also found artifact related volume differences to be seen in cortical volumes and the amygdala. This indicates that cortical volume as well as some subcortical structures might be especially influenced by motion artifacts. Taken together, these findings suggest that the proposed QC rating system is able to identify problematic T1-weighted images [i.e., C3 (fail)] that may otherwise bias sMRI results.

It has to be noted that some other research groups recommend to exclude data from the “moderate” data quality category being C2 (check) in our rating category (Wilke et al., 2002; Shaw et al., 2007; Fjell et al., 2015; Reuter et al., 2015). Still the exclusion of C2 (check) data might not be pragmatically feasible in studies with clinical or developmental populations as it may lead to excluding too many participants. Including images rated C2 (check) and quality controlling their processed data more closely might be more practicable to save valuable data. Importantly, in our study no significant volume differences were found between categories C1 (pass) and C2 (check), which suggests negligible differences between those categories making them both appropriate to include in further statistical analyses.

We did not find differences in motion artifacts between the clinical groups and the TD group. This is in contrast to previous findings (Pardoe et al., 2016) where patient groups moved more than control groups. These differences may be due to our protocol and approach to minimize motion artifacts. First, the research team was more aware of these artifacts, and second, after a thorough qualitative QC, some participants could be measured again. Still even excluding data from only category C3 (fail) holds the risk to fog real group differences. Participants that have to be excluded due to motion artifacts may be the ones suffering from more severe psychopathology and accordingly show more structural alterations. Excluding these participants may thus introduce a selection bias. Therefore, our recommendation is to first train the research team including MR technologists to prepare participants for scanning, to identify motion artifacts directly after scanning and to rescan participants with moderate to strong motion, if possible. Afterwards, data needs to be checked using a detailed rating system as presented here. Taken together, more stringent method reporting in sMRI studies is crucial to guarantee consistent data quality.

Besides retrospective qualitative QC, new approaches to reduce motion like volumetric navigator systems (Tisdall et al., 2016) and other prospective motion correction systems (Brown et al., 2010; Kuperman et al., 2011; Tisdall et al., 2012; Maclaren et al., 2013) have been introduced (see Zaitsev et al., 2015 for a review). However, these techniques are only useful in certain settings, mostly require extra equipment, and are time-consuming or costly. In contrast, our workflow and rating system are easy to adapt, applicable for samples known to show motion during scanning, such as developmental or clinical populations, and require no extra equipment to reduce motion artifacts in subsequent data analysis. Moreover, other visual qualitative QC rating systems have been found to yield similar results as quantitative measures like quantitative motion estimates (Pardoe et al., 2016) and root mean square displacement per minute (RMSpm) has been shown to correlate with QC ratings indicating that manual QC correctly identifies cases with motion (Reuter et al., 2015).

### Limitations

This QC workflow has currently only been applied to a single automated image processing technique (FreeSurfer). However, it is expected that any intensity-based segmentation or classification technique might be affected in a similar way (Blumenthal et al., 2002) and that the QC workflow may be adopted to these techniques as well. It also has only been applied to T1-weighted sMRI images. For further detailed information on sMRI artifacts and examples for QC in T2-weighted images and proton density (PD) weighted images, please see Jones and Marietta (2012). It also has to be noted that the intra-rater reliability of this rating system was not computed. It is advised that future studies investigate both inter-rater and intra-rater reliability. Likewise, all images were rated within a single week. In the case where images must be rated over time should also consider investigating and reporting rater drift. In addition, as our sample was quite small and restricted age wise, more research is needed to apply this QC workflow and rating system to larger datasets (e.g., publically available MR databases) and to more diverse populations (adults, other clinical groups, and other age groups—e.g., younger children). Furthermore, we saw irregularities in skull strip throughout all groups in the detailed automated processing pipeline QC. Though it is not ideal, this was seen as a rather random irregularity and thus data was not excluded based on this factor alone. Finally, even though the proposed QC rating system is able to determine the most problematic T1-weighted images, it must be noted that in-scanner motion might lead to biases in anatomical estimations, even at levels which do not manifest in visible motion artifacts (Alexander-Bloch et al., 2016).

## Conclusion

We provide a standard hands-on workflow and qualitative QC rating system to help minimizing biases in results produced by motion artifacts. The application will help researchers improve the quality of future sMRI studies.

## Ethics statement

Ethikkommission an der Technischen Universitaet Dresden, Germany (EK 293092010). Both participants and parents or legal guardians respectively gave their written informed consent prior to participation. Minors as well as their parents or legal guardians respectively received detailed information about the study procedure. It was made sure all minor participants understood the study procedure and experiments which were carried out.

## Author contributions

MS and VR contributed to the experimental design of the study. Data acquisition was carried out by NV, JB, and LB. Data analysis was performed by LB, and NV. LB, MH, and NV were involved in the interpretation of data. The manuscript was drafted by NV and LB. All authors revised the manuscript critically, approved the submitted version to be published and hold themselves accountable for all aspects of the work in ensuring that questions related to the accuracy or integrity of any part of the work are appropriately investigated and resolved.

### Conflict of interest statement

The authors declare that the research was conducted in the absence of any commercial or financial relationships that could be construed as a potential conflict of interest.
