# Supplementary material for: Quality Control of Structural MRI Images Applied Using FreeSurfer—A Hands-On Workflow to Rate Motion Artifacts
Source: Front Neurosci. 2016 Dec 6;10:558. doi: 10.3389/fnins.2016.00558 (PMC5138230; doi:10.3389/fnins.2016.00558)
Supplement: Supplementary file 1 [file Table1.pdf]

Supplementary Material

Quality control of structural MRI images applied using FreeSurfer - a hands-on workflow to rate motion artifacts

Lea L. Backhausen[1], Megan Herting[2], Judith Buse[1], Veit Roessner[1], Michael N. Smolka[3], Nora C. Vetter[1*]

*** Correspondence:** Nora C. Vetter, nora.vetter@tu-dresden.de

1 Overview of relevant sMRI artifacts

Type of artifact	Description
Motion	Periodic motion like respiratory and cardiac motion or vascular flow causes coherent ghost structures, whereas aperiodic motion cause blurring of the image (Wood & Henkelman, 1985a). The latter includes e.g. swallowing, blinking, chewing, turning, fidgeting or repositioning a limb (Bellon et al., 1986) which is more substantial in head/brain imaging. Motion always leads to ghosting or blurring in the phase encoding direction for subject motion is slower than the fast sampling process along frequency encoding direction (Gallagher, Nemeth, & Hacein-Bey, 2008; Stadler, Schima, Ba-Ssalamah, Kettenbach, & Eisenhuber, 2006).
Ringing (Gibbs phenomenon)	The Gibbs phenomenon causes ringing or truncation artifacts. They appear in rings of periodic nature parallel to edges of abrupt intensity change, e.g. skull/ brain (Wood & Henkelman, 1985b), and fade out with more distance from these borders. This is the result of imperfect approximation of sharp edges by Fourier transform (Gallagher et al., 2008). This artifact should be handled with care for it is easily confused with ghosting caused by motion (Bellon et al., 1986). According to Stadler and colleagues (2006) ringing truncation artifacts can be minimized by increasing acquisition matrix to 256 or larger and/ or smaller FOV.

Susceptibility phenomenon	Ferromagnetic objects like prostheses, implants, safety pins, hair pins, bra stays, dental braces, jewelry or mascara have higher magnetic susceptibility, meaning the property of matter of becoming magnetized when exposed to a magnetic field (Stadler et al., 2006), relative to tissue (Bellon et al., 1986). This produces localized field changes which results in bright and dark areas with spatial distortion of surrounding anatomy (Stadler et al., 2006).

References

Bellon, E., Haacke, E., Coleman, P., Sacco, D., Steiger, D., & Gangarosa, R. (1986). MR artifacts: a review. *American Journal of Roentgenology*, *147*(6), 1271–1281. http://doi.org/10.2214/ajr.147.6.1271

Gallagher, T. A., Nemeth, A. J., & Hacein-Bey, L. (2008). An introduction to the fourier transform: Relationship to MRI. *American Journal of Roentgenology*, *190*(5), 1396–1405. http://doi.org/10.2214/AJR.07.2874

Stadler, A., Schima, W., Ba-Ssalamah, A., Kettenbach, J., & Eisenhuber, E. (2006). Artifacts in body MR imaging: their appearance and how to eliminate them. *European Radiology*, *17*(5), 1242–1255. http://doi.org/10.1007/s00330-006-0470-4

Wood, M. L., & Henkelman, R. M. (1985a). MR image artifacts from periodic motion. *Medical Physics*, *12*(2), 143–151. http://doi.org/10.1118/1.595782

Wood, M. L., & Henkelman, R. M. (1985b). Truncation artifacts in magnetic resonance imaging. *Magnetic Resonance in Medicine*, *2*(6), 517–526. http://doi.org/10.1002/mrm.1910020602
