# Supplementary material for: Quality Control of Structural MRI Images Applied Using FreeSurfer—A Hands-On Workflow to Rate Motion Artifacts
Source: Front Neurosci. 2016 Dec 6;10:558. doi: 10.3389/fnins.2016.00558 (PMC5138230; doi:10.3389/fnins.2016.00558)

TECHNISCHE UNIVERSITÄT DRESDEN

Faculty of Medicine Carl Gustav Carus, TU Dresden, Germany:
Department of Psychiatry and Neuroimaging Center and Department of Child and Adolescent Psychiatry

QC rating system for T1-weighted images

# - example images -

DRESDEN concept
Exzellenz aus Wissenschaft und Kultur

Rating system structure

**Step 1**<br>**Image sharpness**	R1 (good): Clear/ rather clear image; ghosts, blurred regions or other artifacts if at all minor; no susceptibility artifacts<br>R2 (moderate): Rather coarse/ blurred image; moderate motion artifacts; if susceptibility artifacts are present they do not influence relevant areas<br>R3 (bad): Obviously coarse/ blurred image; major motion and susceptibility artifacts (e.g. due to dental braces)
**Step 2**<br>**Ringing**	R1 (good): No/ slight ringing artifacts seen; at most in one region<br>R2 (moderate): Ringing artifacts in more than one region<br>R3 (bad): Circular ringing artifacts throughout the whole image
**Step 3**<br>**Contrast to noise ratio (subcortical structures)**	R1 (good): Sharp edges; structures can be well identified<br>R2 (moderate): Structures still can be identified but less clear<br>R3 (bad): Structures can hardly be identified
**Step 4**<br>**Contrast to noise ratio (gray matter and white matter)**	R1 (good): Sharp edges; gray matter and white matter are well differentiated<br>R2 (moderate): gray matter and white matter not well differentiated<br>R3 (bad): Borders of gray matter and white matter blend; not differentiated at all

Rating system T1-weighted images

TECHNISCHE UNIVERSITÄT DRESDEN

Rating system structure

1. **Each image is rated R1 – R3 on each of the four steps according to the criteria listed above**

2. **These four ratings of each step are then merged into a mean score which represents the final category → C1 (pass), C2 (check) or C3 (fail)**

- Depending on the study's focus, the different steps can be weighted when calculating the average; such as step 3 of subcortical structures could have a higher impact on the final score/category

- These final category assignments can be used to decide whether to include or exclude images from further analyses
  - **C1 (pass):** include or exclude after quick automated processing pipeline QC
  - **C2 (check):** include or exclude after detailed automated processing pipeline QC
  - **C3 (fail):** exclude from further data analysis

Examples **Category C1 (pass)**

Step 1
Image sharpness: R1 (good)

Clear/ rather clear image; ghosts, blurred regions or other artifacts if at all minor; no susceptibility artifacts

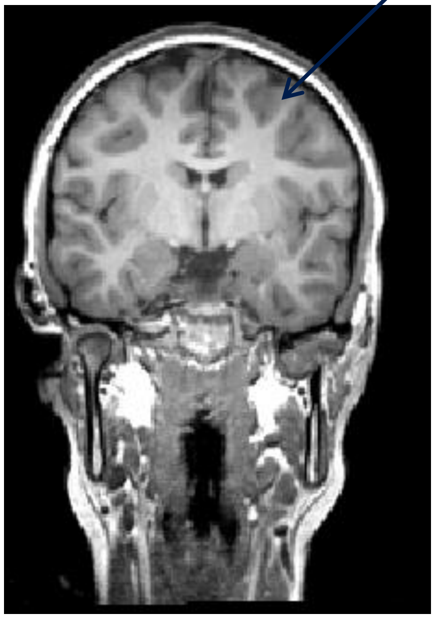
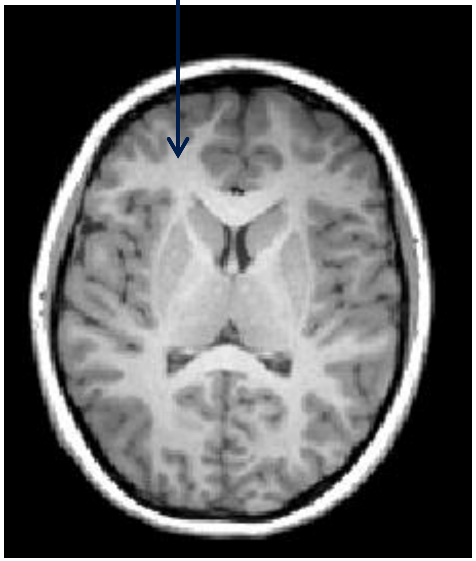
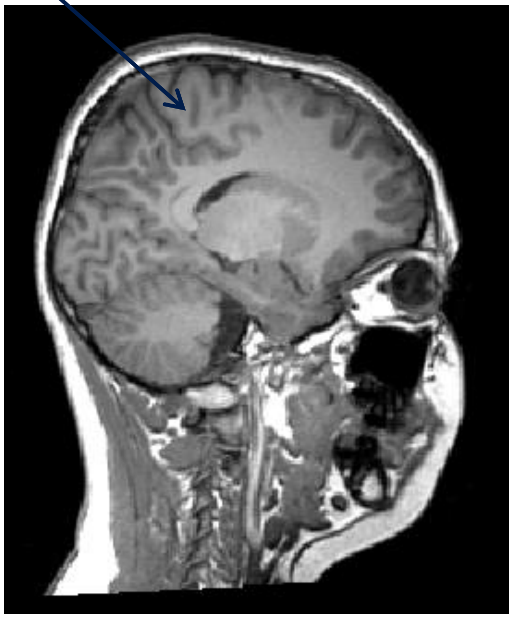

Examples **Category C1 (pass)**

Step 2
Ringing: R1 (good)

Ringing in one region

No ringing in other regions

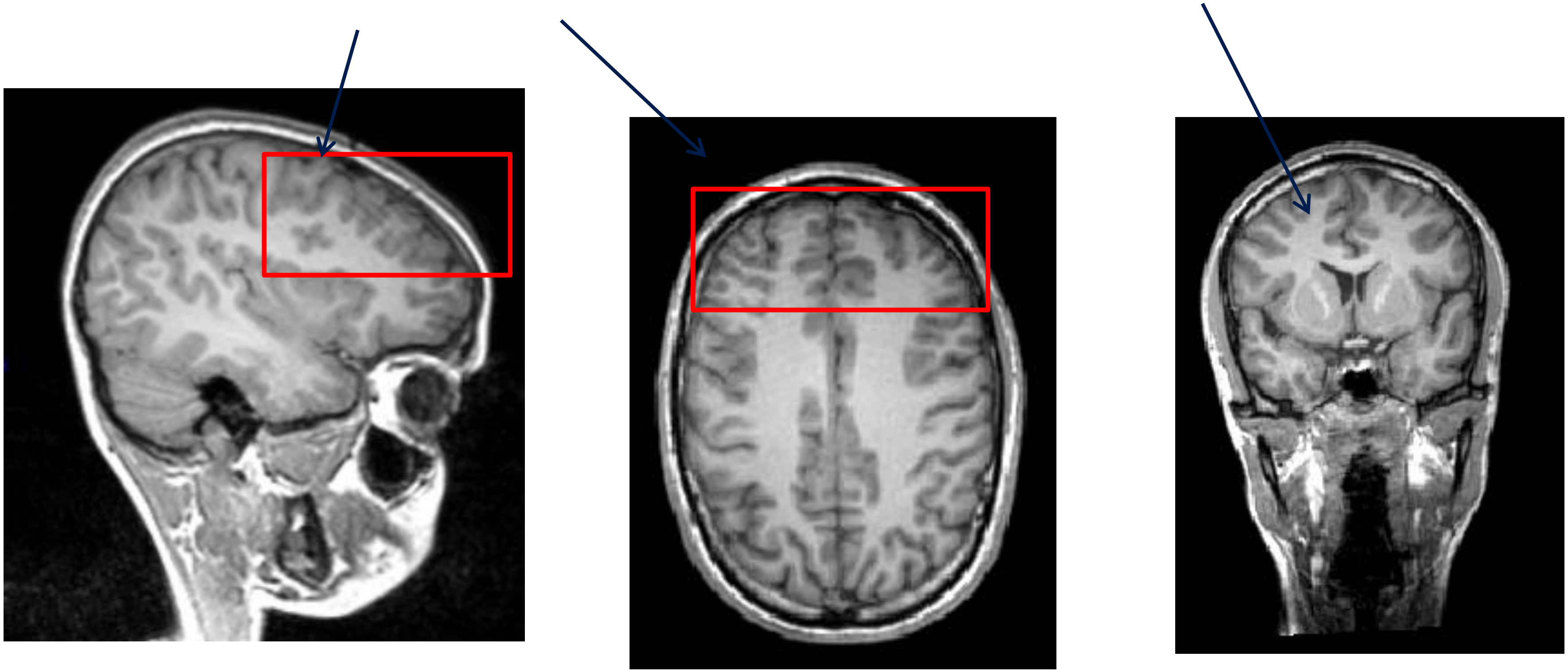

Examples **Category C1 (pass)**

Step 3
CNR (subcortical structures): R1 (good)

Structures can be well identified, sharp edges

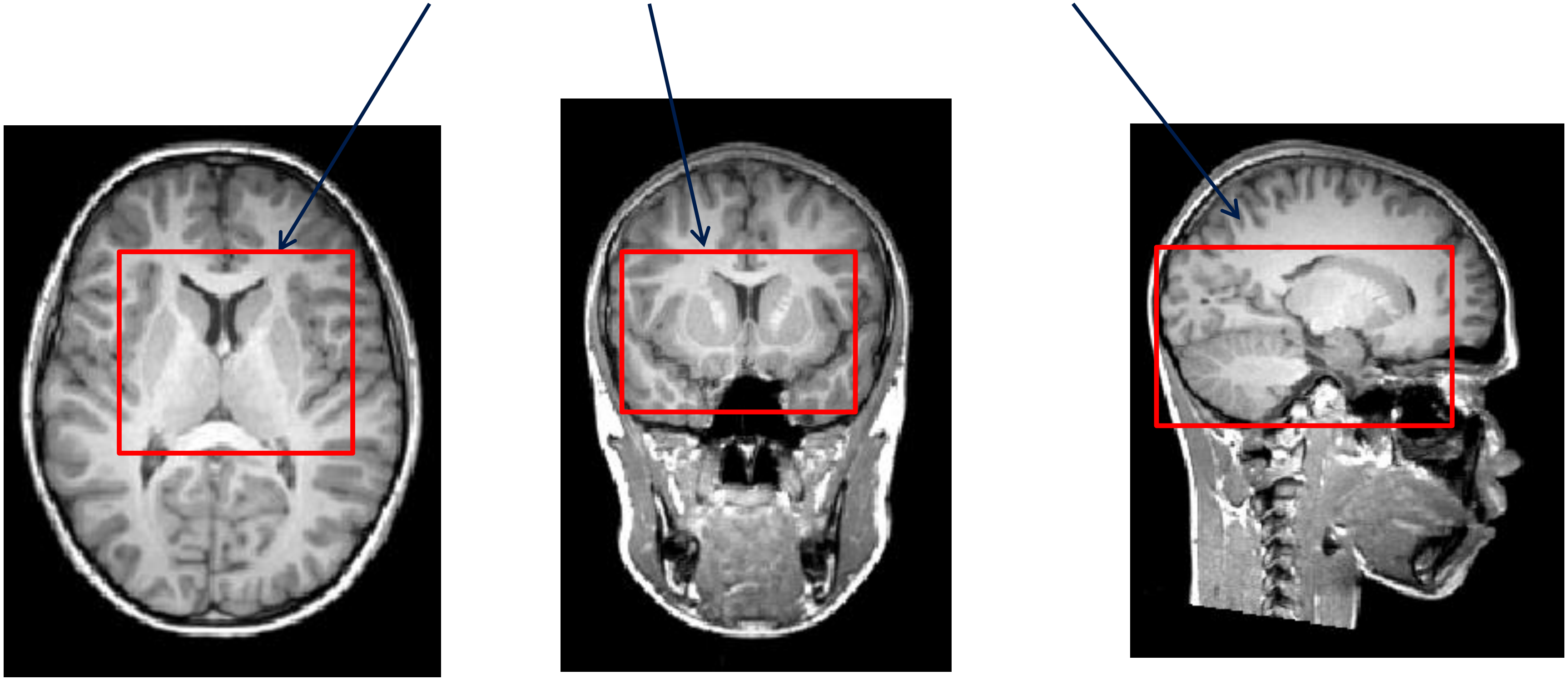

Examples **Category C1 (pass)**

Step 4
CNR (gray matter and white matter): R1 (good)

Gray matter and white matter are well differentiated

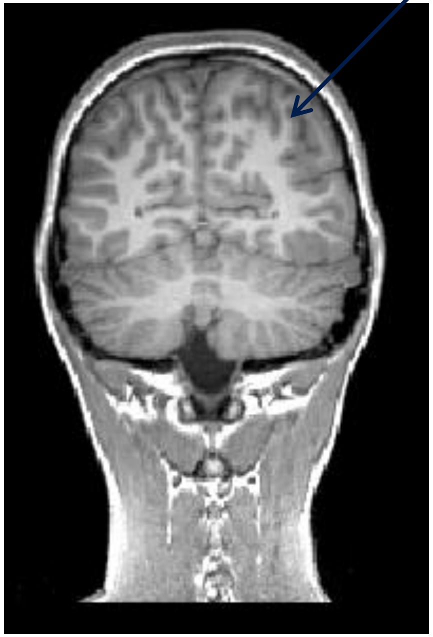

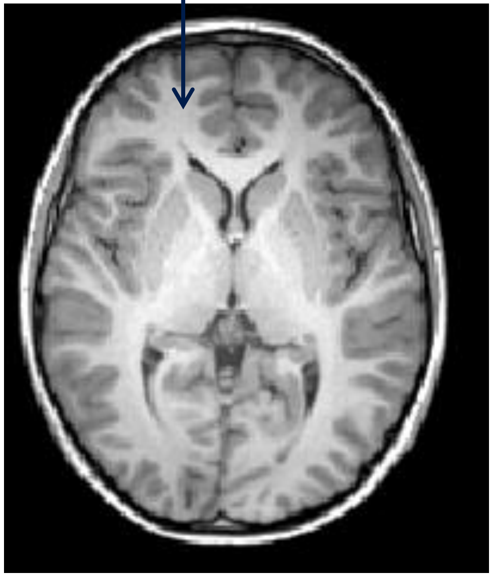

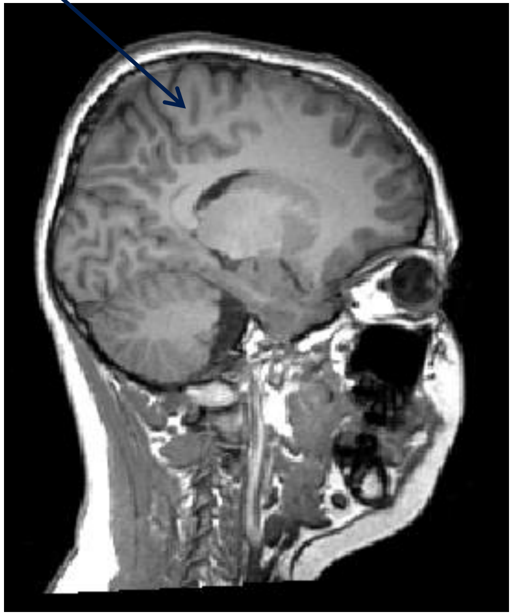

Examples **Category C2 (check)**

Step 1
Image sharpness: R2 (moderate)

Straight-lined distortion but image not much affected

blurred jaw/palate, may indicate shoddy image quality

Image looks quite coarse/ washy

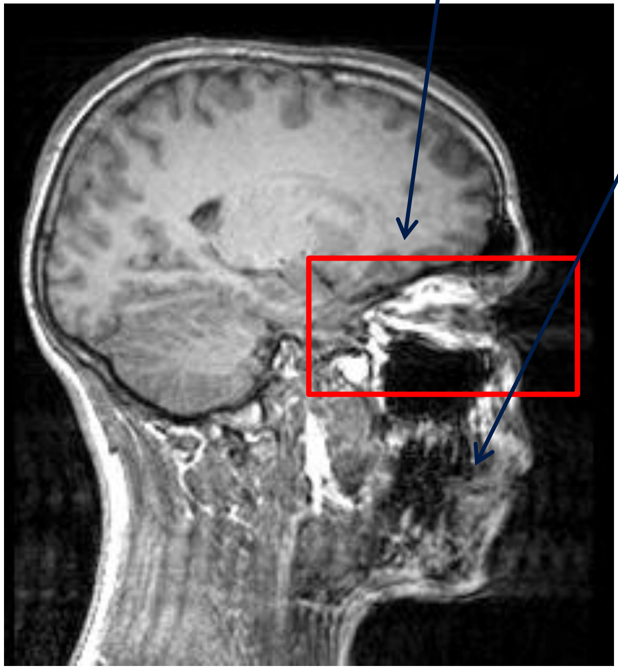

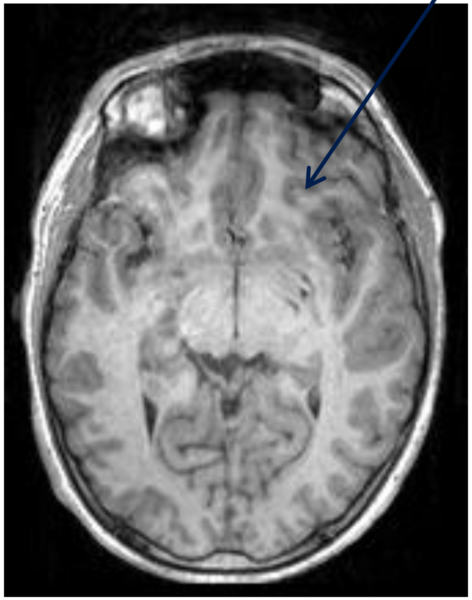

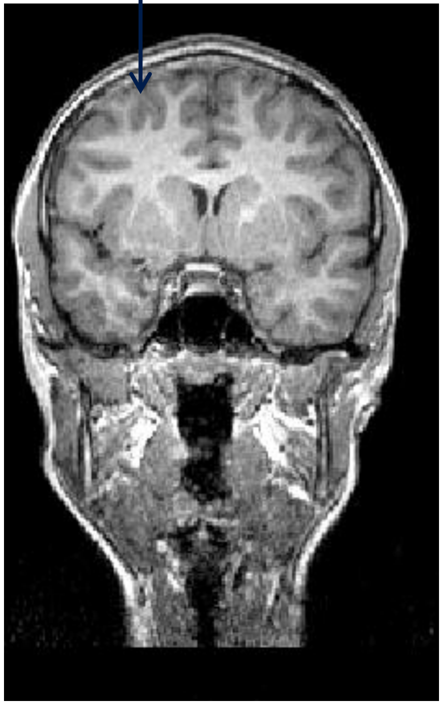

Examples **Category C2 (check)**

Step 2
Ringing: R2 (moderate)

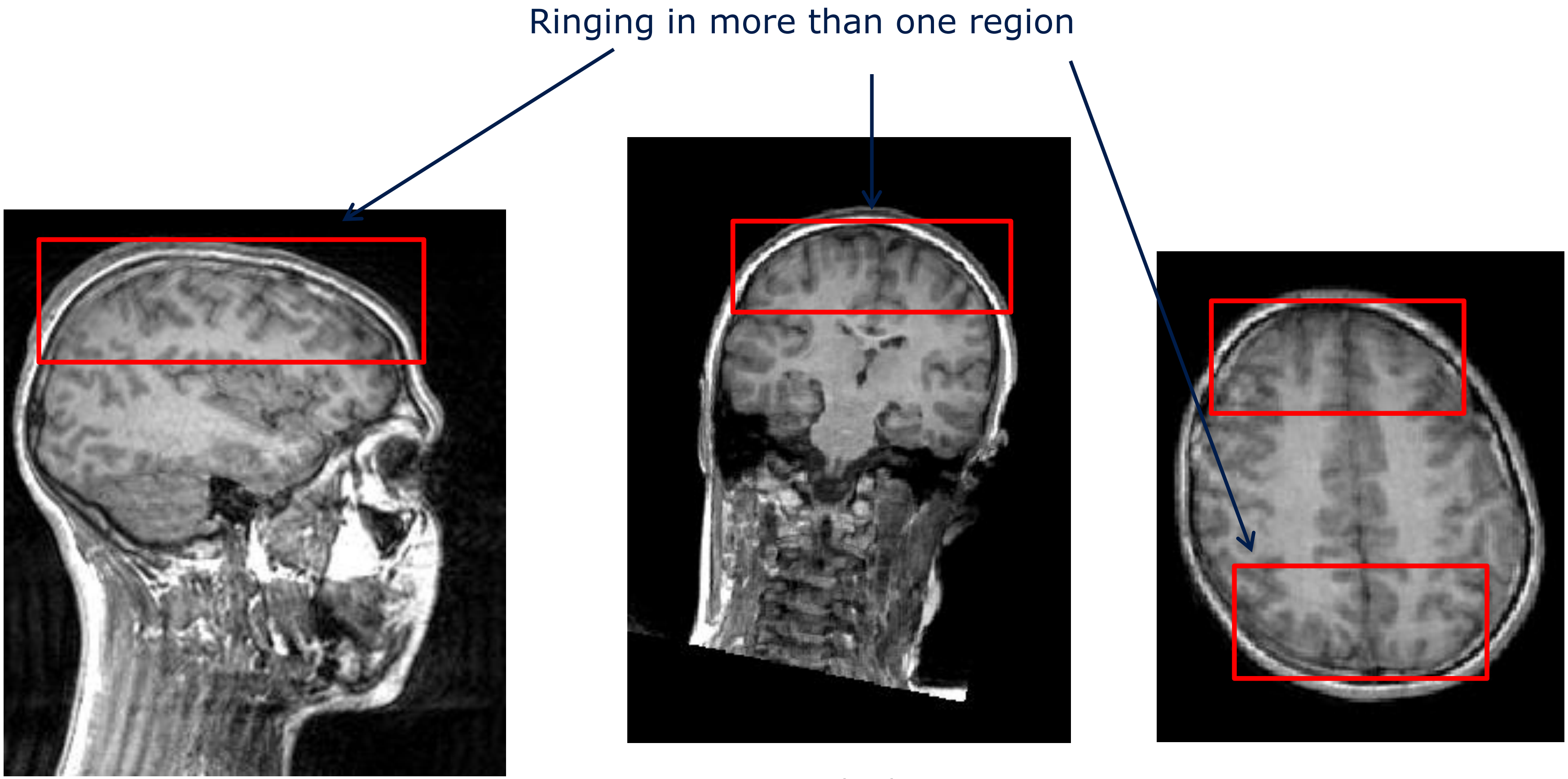

Examples **Category C2 (check)**

Step 3
CNR (subcortical structures): R2 (moderate)

Structures can be identified but less clear (coarse image)

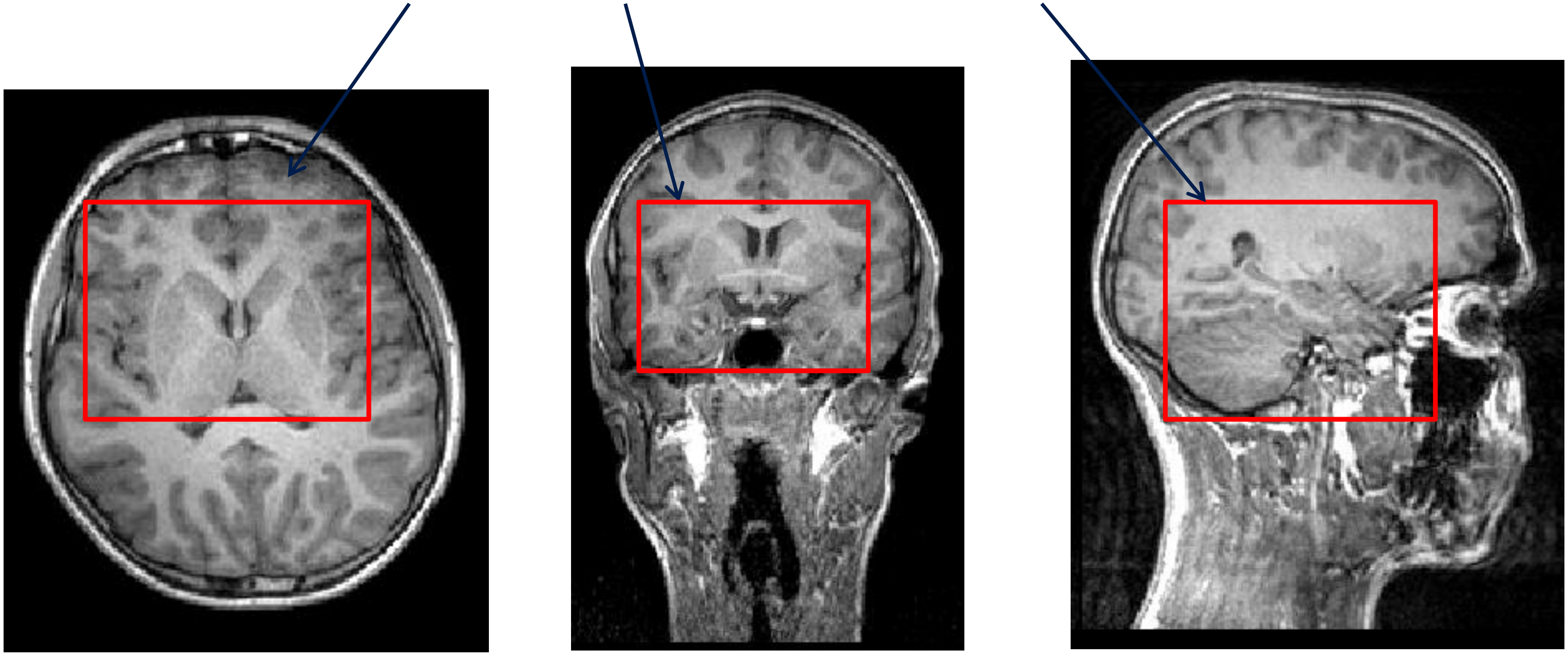

Examples **Category C2 (check)**

Step 4
CNR (gray matter and white matter): R2 (moderate)

gray matter and white matter not well differentiated but seems okay

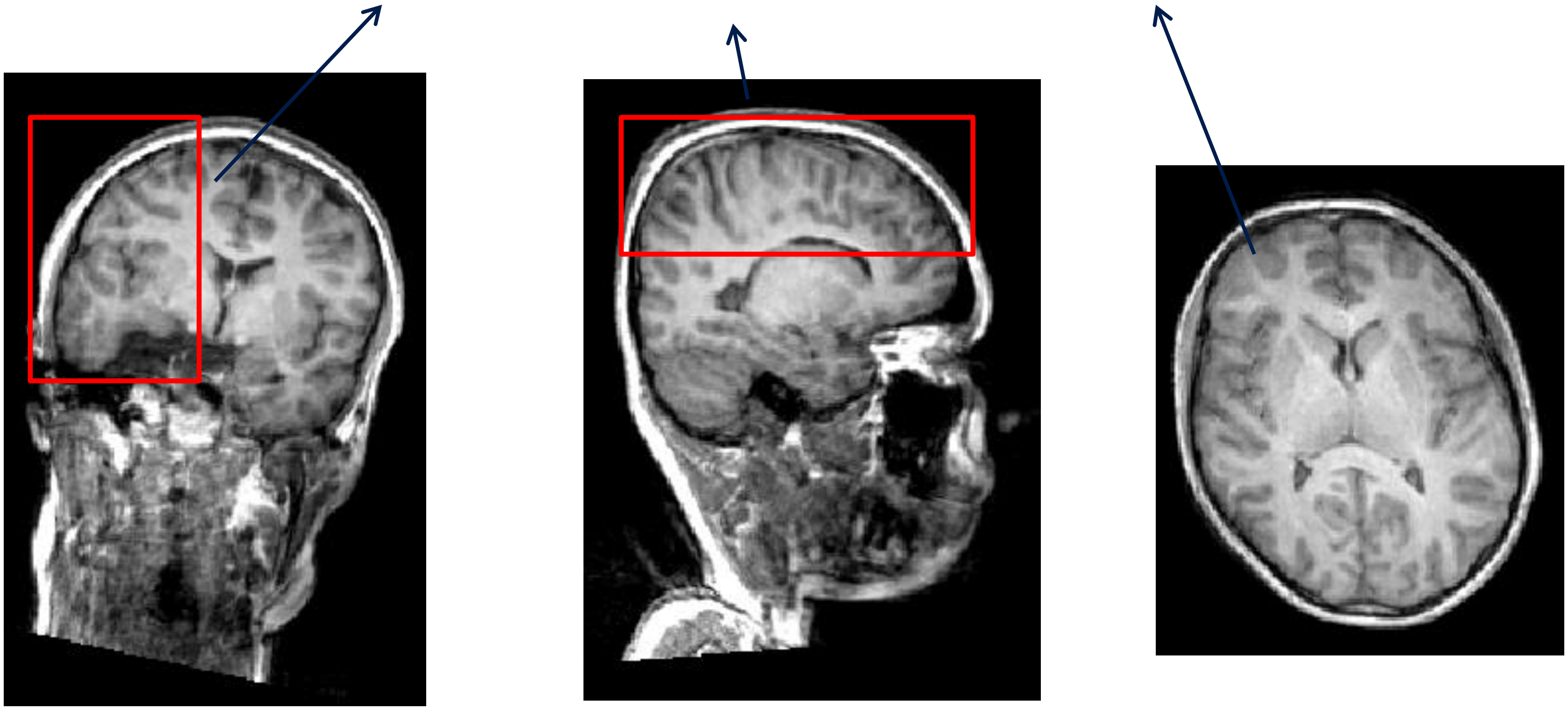

Examples **Category C3 (fail)**

Step 1
Image sharpness: R3 (bad)

Coarse image, severely blurred jaw/palate

Major erasement (dental brace), frontal cortex „eroded“ and severe straight-lined distortion

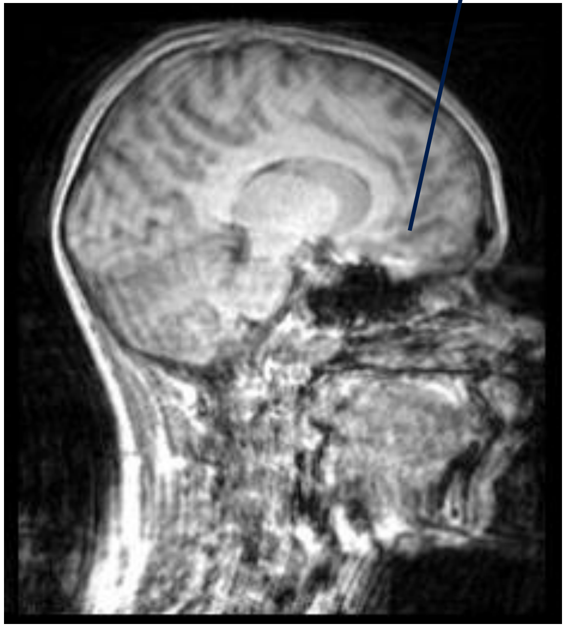

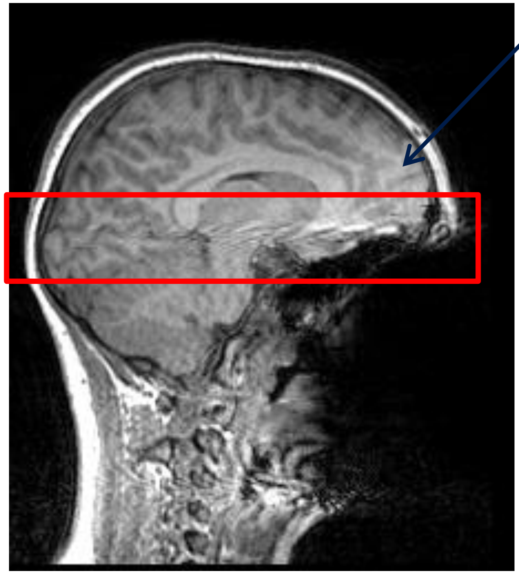

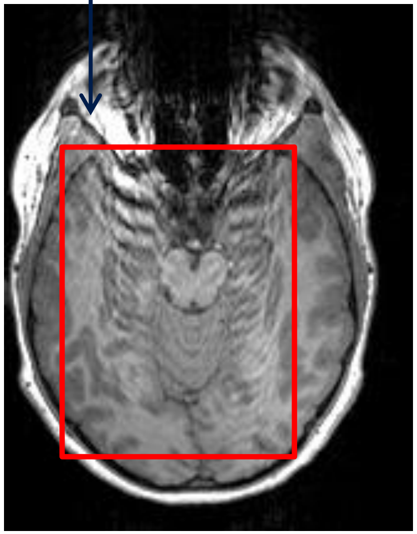

Examples **Category C3 (fail)**

Step 2
Ringing: R3 (bad)

Severe ringing, almost circular

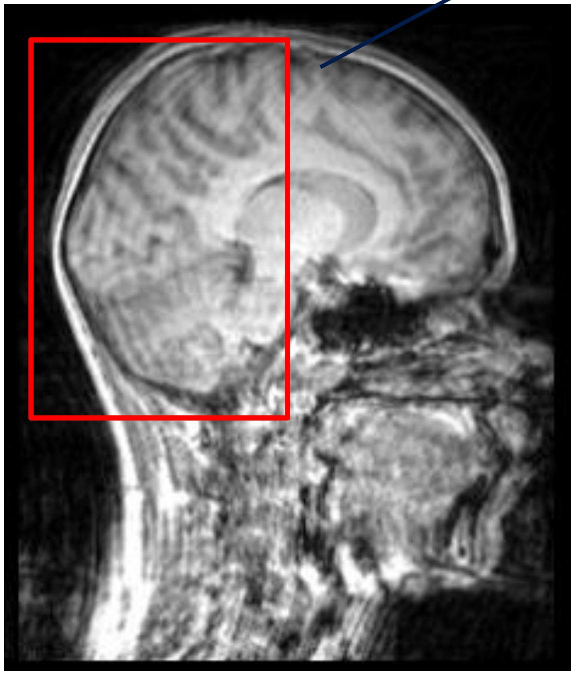

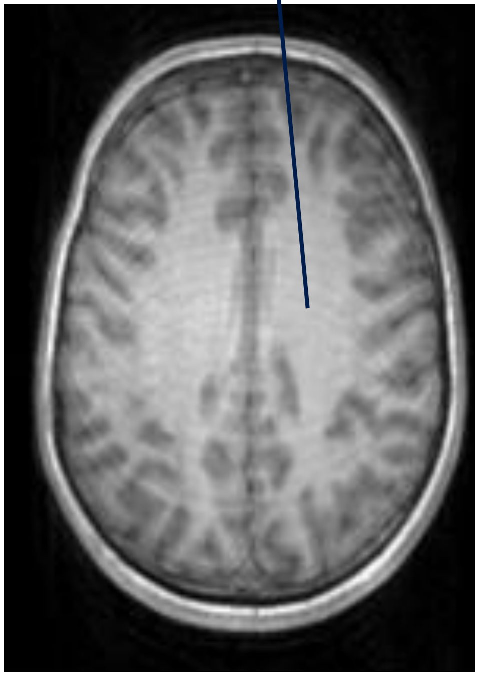

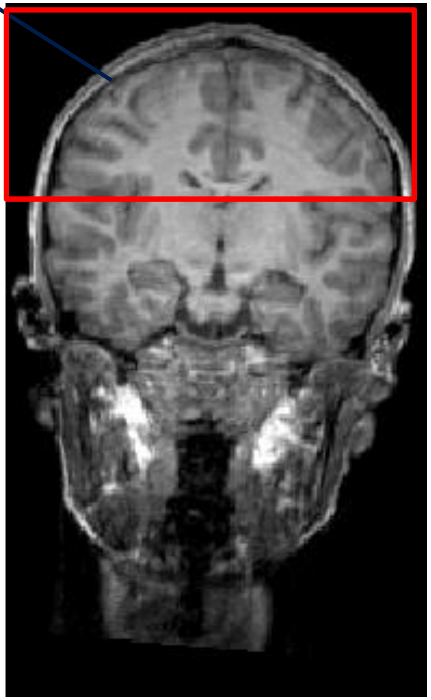

Examples **Category C3 (fail)**

Step 3
CNR (subcortical structures): R3 (bad)

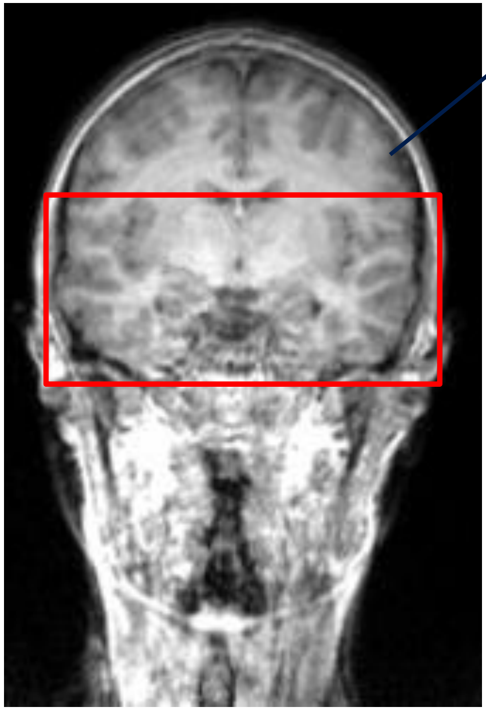

Subcortical structures can hardly be identified

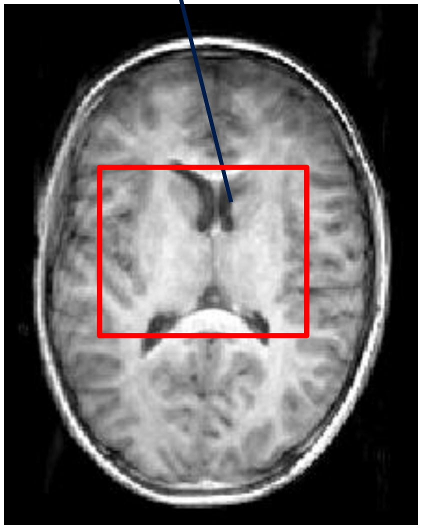

Identifiability of subcortical structures is strongly limited

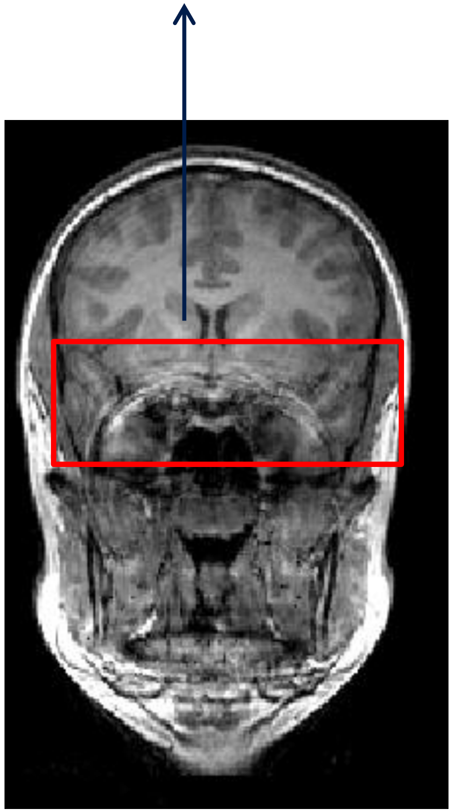

Examples **Category C3 (fail)**

Step 4
CNR (gray matter and white matter): R3 (bad)

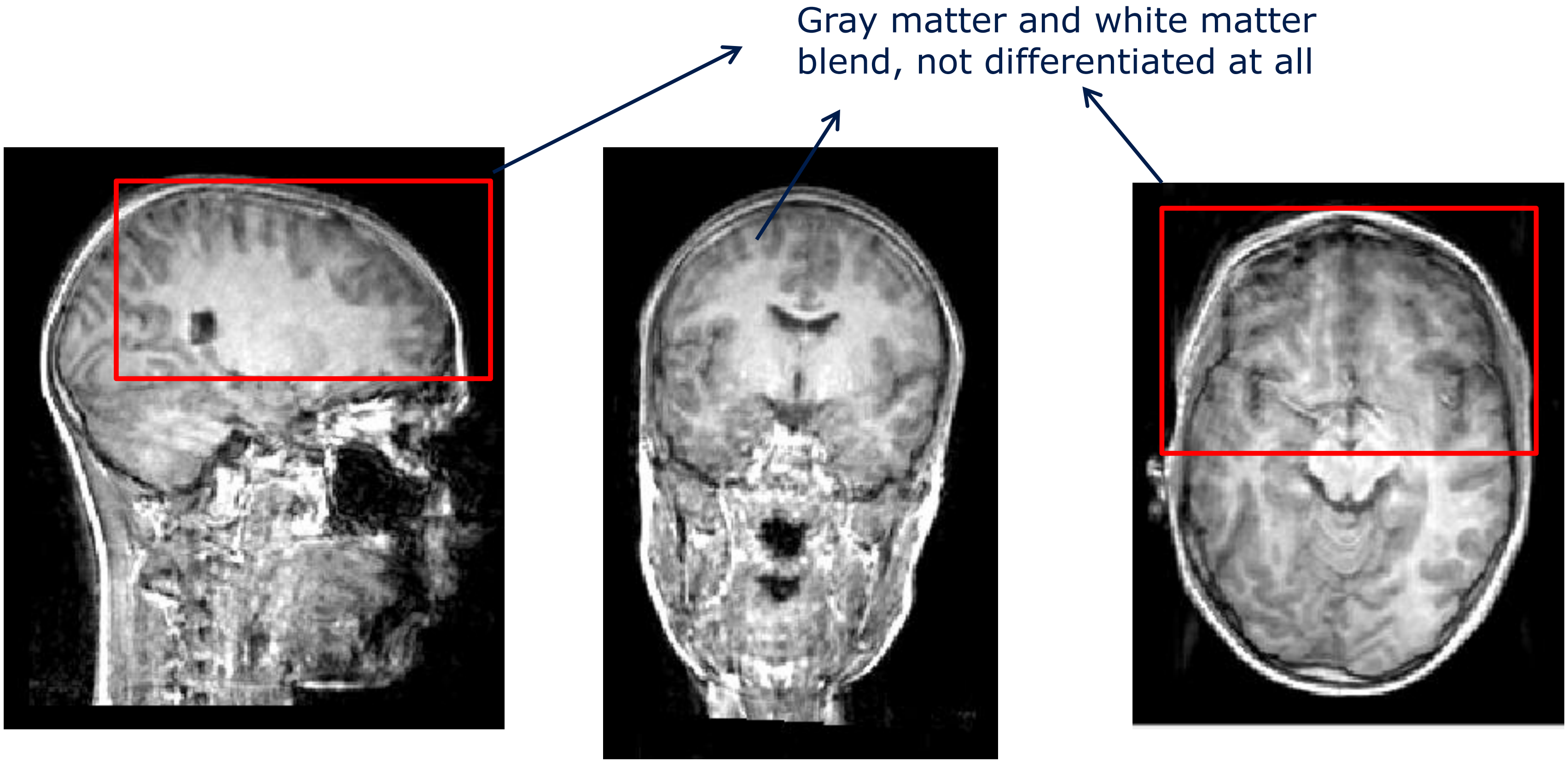

Supplement: Supplementary file 2 [file Presentation1.PDF]
